# Case of Disease of the Thyroid Gland

**Published:** 1828-11

**Authors:** Congreve Selwyn

**Affiliations:** Member of the Royal College of Surgeons, London.


### THYROID GLAND.

*Case of Disease of the Thyroid Gland.* By Congreve Selwyn, Member of the Royal College of Surgeons, London.

Thomas Stephens, ætatis fourteen, farm-servant to Mr. Cummins, of Dymock, in the county of Gloucester, was admitted a patient of the Ledbury Dispensary, to which I am surgeon, for an enlargement of the right side of the thyroid gland, which had been of eight years' standing, and which very much interfered with respiration, particularly on exertion. My friend Mr. Thomas Blizard happening to be at my house, I requested his opinion. On examination, it was found that the right side of the gland was converted into a thick cyst containing fluid, and it was agreed that I should puncture the swelling with a small trocar and canula; which I did in the presence of Mr. Blizard, and drew off six ounces of fluid by measure.

The lad was desired to attend again in a week, as we anticipated the sac would refill; and we agreed, if it did, to evacuate it again, and pass a small seton across the cyst. The patient attended as he was desired, and I found the swelling had acquired nearly the same magnitude as at first, and, on re-evacuating its contents, nearly the same quantity of fluid had been secreted in the short space of a few days. I now passed the seton through the sac, and left it with a view to induce suppuration in it and its obliteration.

The boy wore the seton for several weeks, during which a copious suppuration was kept up; but at length, the matter lodging, and being in part absorbed into the system, and causing constitutional irritation, I laid the cavity open, (first withdrawing the seton,) with a director and bistoury, to the extent of an inch and a half; and, on passing my finger into the wound, I found the cyst partly ossified. The case went on very well, the cavity gradually filling up, and the whole cyst becoming completely obliterated.

The lad has continued perfectly well ever since, which is now a year and a half ago.

I have since treated three other cases of the same description in a similar way, and with equal success.

One of these, in particular, deserves notice, as the constitution of the boy was in a very bad state: he had been in the Glocester Infirmary for disease of the lungs and tendency to dropsy; the swelling in the neck co-existing at the same time. Whether these symptoms were caused by or connected with the disease in the throat, I cannot take upon me to say: however, they yielded to the local and constitutional treatment pursued by me, and he is now a strong hearty young man.

*Ledbury, Herefordshire.*

